# Biofunctional Pharmaceutical Additives for Targeted, Improved Bioavailability and Safety of Medicine

**DOI:** 10.3390/pharmaceutics17080978

**Published:** 2025-07-28

**Authors:** Hamdy Abdelkader, Adel Al Fatease, Zimei Wu

**Affiliations:** 1 Department of Pharmaceutics, College of Pharmacy, King Khalid University, Abha 62529, Saudi Arabia; afatease@kku.edu.sa; 2 School of Pharmacy, The University of Auckland, Auckland 1010, New Zealand; z.wu@auckland.ac.nz

## 1. Introduction

For many decades, conventional pharmaceutical excipients have been used to optimize the palatability, processing ability, flowability, and compressibility of various types of medication throughout the production process. Examples of these excipients include lubricants, glidants, binder, diluents, and disintegrants. Various additional pharmaceutical components and excipients have been employed to increase the chemical, physical, and microbiological stability of different types of medication. However, numerous drug–excipient incompatibilities; excipient-related adverse effects, including local irritation, interference at the absorption sites due to g-glycoproteins and efflux pumps; and other systemic toxicities have been documented in the literature [1,2,3]. Recent developments in medical and pharmaceutical sciences have led to new therapeutic agents with poor biopharmaceutical qualities, such as limited permeability and low solubility, being introduced to the market. Suboptimal efficacy and unpleasant reactions to medication might occur as a result of erratic drug absorption, irritation, poor pharmacokinetic profiles, and lack of tissue targeting [4].

Excipients or additives have traditionally been considered biologically inactive. In recent years, however, biofunctional excipients have emerged, including lipids, smart polymers, and other natural and safe additions that can change a drug’s permeability, pharmacokinetic profiles, release properties, and solubility. Typical examples include amino acids, cyclodextrins, in situ-forming polymers, smart polymers, etc., and various biomimetic materials, such as cell-based systems, have also emerged as promising delivery enhancers [5,6,7,8,9].

In light of these factors, this Special Issue (SI), “Biofunctional Pharmaceutical Additives for Targeted, Improved Bioavailability and Safety of Medicine”, focuses on recent advancements in biofunctional additives and excipients. These developments are used to create pharmaceutical formulations and delivery systems (for topical or systemic routes) that may provide targeted drug delivery or modified drug release to improve efficacy and lessen irritation at the absorption site and beyond [1].

This SI encompasses nine original research papers and a review article and covers a wide range of creative and adaptable research topics. It highlights developments related to the discovery of excipients, e.g., amino acids, cyclodextrins, hydrocolloid polymers, and molecular targets and microneedle systems to create safer medications and more effective sustained release systems. The ten publications were contributed by 72 researchers across the globe (Asia, Europe, Africa, North America, and Australasia), offering significant insights into the pharmaceutical additives used in a range of drug-delivery systems and potentially contributing to better clinical outcomes.

## 2. Overview of the Published Articles

Sarkhel et al.’s review features various nanocarriers that can be used to improve the biopharmaceutical characteristics of antidiabetic drugs. Diabetes mellitus is a major health issue that is on the rise worldwide. Nanomaterials have displayed considerable potential for advancing the delivery of antidiabetic medications, as well as in the realms of diagnosis, glucose sensing, and glucose monitoring. Liposomes, solid lipid nanoparticles (SLNs), polymeric nanoparticles, nanostructured MOFs, quantum dots, DNA origami, and other nanotechnology-based infrastructure innovations all provide numerous advantages, such as increased bioavailability, targeted delivery, controlled release, enhanced stability, dose proportionality, decreased toxicity, and decreased dosing frequency (Contribution 1).

In an innovative leap toward multifunctional wound therapy, this paper introduces a groundbreaking oral drug-delivery system that combines traditional methods with solid lipid nanoparticles (SLNs) loaded with Marshmallow extract and clove oil and embedded in a collagen sponge. This unique hybrid platform not only enhances the solubility and stability of natural bioactive substances but also offers controlled, sustained drug release and synergistic antimicrobial and healing effects specifically tailored to target diabetic oral ulcers. With remarkable optimization (particle size 110 nm, entrapment efficiency 90%, zeta potential −24 mV), the formulation showed superior in vivo wound healing, robust antibacterial and antifungal activity, and biocompatible integration into the oral mucosa. The inclusion of collagen sponge provided structural support and prolonged retention, making this system a clinically promising, natural solution that can overcome the limitations of traditional ulcer treatments and will pave the way for next-generation bioactive wound care (Contribution 2).

One of the most prevalent naturally occurring polysaccharides is chitosan, which is mostly obtained from marine crustaceans. When used in the formulation of drug delivery nanoparticles, chitosan offers numerous benefits, such as biocompatibility and biodegradability. Furthermore, it possesses mucoadhesive qualities due to the positive charge on its surface, making it a viable option for ocular medication administration. Osi et al. studied the ocular absorption pathway of Fasudil hydrochloride (a promising antiglaucoma drug) using an ex vivo model. Chitosan nanoparticulate systems were prepared using the ionic gelation method, and they conducted in vitro release and ex vivo permeation studies of Fasudil obtained via the prepared chitosan nanoparticles. Finally, the systems’ ocular (conjunctival and corneal) tolerability were assessed (Contribution 3).

Mubeen et al. conjugated linoleic acid–carboxymethyl chitosan polymeric micelles for the oral delivery of paclitaxel (PCL). The developed micelles were evaluated by particle size, zeta potential, Fourier transform infrared spectroscopy (FTIR), differential scanning calorimetry (DSC), and thermogravimetric analysis (TGA). When PCL was contained within micelles, its solubility increased by almost 13.65 times (around 60 µg/mL). The outcomes demonstrated that PCL’s solubility and pharmacokinetics improved in the micelles of the LA-CMCS conjugate. Pharmacokinetic investigations revealed that compared with PCL suspension, LA-CMCS micelles loaded with PCL exhibited significantly better oral absorption. Overall, this study concludes that amphiphilic CMCS analogues could serve as an effective drug-delivery system for boosting the biopharmaceutical effectiveness of drugs with weak water-solubility (Contribution 4).

Elhabal et al. pioneered a dual-technology approach that marks a breakthrough in ocular therapeutics by engineering dissolving microneedles loaded with lactoferrin nanosuspensions (BLF-NS-MNs) for the treatment of dry eye disease (DED). Leveraging the synergistic power of nanotechnology and microneedle delivery, the optimized BLF nanosuspensions (215 nm, EE% 90%) were integrated into biodegradable PVP/HPMC microneedles to overcome key challenges in ocular drug delivery, such as low bioavailability, poor permeability, and rapid drainage. The resulting formulation demonstrated exceptional corneal penetration, sustained drug release (95% over 24 h), and robust anti-inflammatory activity, as evidenced by the near-normalization of critical inflammatory biomarkers (TNF-α, IL-6, MMP-9, IL-1β, MCP-1). Furthermore, the BLF-NS-MNs showed superior antimicrobial activity against resistant pathogens like MRSA and Aspergillus Niger, outperforming standard antibiotics. This work not only delivers a highly biocompatible, non-invasive, and pain-free therapy but also sets the stage for next-generation smart ocular drug systems tailored to treat chronic inflammatory eye conditions (Contribution 5).

Dasatinib (DAS), a potent anticancer drug, has been subjected to formulation enhancements to address its significant first-pass metabolism, poor absorption, and limited oral bioavailability. To improve its release profile, DAS was embedded in a matrix of the hydrophilic polymer polyvinylpyrrolidone (PVP). Drug amorphization was induced in a planetary ball mill by solvent-free co-grinding, facilitating mechanochemical activation. This process resulted in the formation of amorphous solid dispersions (ASDs). ASD capsules exhibited a notable enhancement in the release rate of DAS compared to capsules containing the initial drug (Contribution 6).

Simvastatin (SVA) is commonly prescribed to treat cardiovascular and hypercholesterolemia. Due to its extensive hepatic first-pass metabolism and poor solubility, its oral bioavailability is 5%. Solid lipid nanoparticles (SLNs) and hydrogel-coated SLNs were investigated as options for overcoming the limited bioavailability of SVA. Four different lipids used alone or in combination with two stabilizers were employed to generate thirteen SLNs. Two concentrations of chitosan (CS) and alginate (AL) were coating materials. SLNs were studied to determine their particle size, zeta potential, in vitro release, rheology, and bioavailability. The viscosities of both the bare and coated SLNs exhibited shear-thinning behavior. In vivo studies illustrated that F11 had the highest plasma concentration compared with the SVA suspension and coated chitosan (F11 (Chitosan 1%)). The greater bioavailability was measured by determining the (AUC) compared that of uncoated samples. The AUCs for F11, F11-CS 1%, and the SVA suspension were 1880.4, 3562.18, and 272 ng·h/mL, respectively. Both bare and coated SLNs exhibited a significantly higher relative bioavailability compared to that from the control SV (Contribution 7).

Olmesartan medoxomil (OLM) is the prodrug of olmesartan, an angiotensin II type 1 receptor blocker with antihypertensive and antioxidant activities and renal protective properties. It exhibits low water solubility, which leads to poor bioavailability and limits its clinical potential. To improve the solubility of OLM, a host–guest inclusion complex (IC) between heptakis (2,6-di-O-methyl)-β-cyclodextrin (DMβCD) and the drug substance was obtained. Along with active substances, excipients play a crucial role in the quality, safety, and efficacy of pharmaceutical formulations. Therefore, the compatibility of OLM/DMβCD IC with several pharmaceutical excipients was evaluated. The results of this study emphasize that OLM/DMβCD IC stands out as a valuable candidate for future research in the development of new pharmaceutical formulations, in which precautions should be considered in choosing magnesium stearate and α-lactose monohydrate as excipients if the manufacture stage requires temperatures above 100 °C (Contribution 8).

Aeonium arboreum extract exhibited strong antioxidant and α-glucosidase inhibitory activities, especially in its 50% Diaion fraction and quercetin-3-rhamnoside, suggesting that it has potential as a safe, natural therapeutic for managing diabetes and metabolic syndrome through multiple biochemical pathways (Contribution 9).

In addition to the research on synthetic biofunctional materials and their applications discussed above, this SI also features an insightful study by Wu’s team, showcasing the development of biomimetic materials—specifically extracellular vesicle (EV)-based drug-delivery systems, an emerging area of interest in biofunctional materials. Despite growing enthusiasm for using EVs in intracellular drug delivery, our understanding of their interactions with cells remains limited, particularly in the case of large EVs (lEVs > 200 nm). Moreover, the low production yields of EVs hinder translational applications. Using pancreatic cancer cell-derived EVs as a model, the team compares the endocytic pathways and uptake mechanisms of small EVs (sEVs < 200 nm, mostly exosomes) and lEVs, revealing distinct internalization routes such as clathrin- and caveolin-mediated endocytosis for sEVs, and actin-dependent phagocytosis/macropinocytosis for both types. Importantly, the study highlights the biofunctional potential of lEVs and demonstrates that the Integra CELLine bioreactor significantly enhances EV production, achieving up to 9-fold higher yields and improved protein content, compared to conventional method based on flasks. These findings not only broaden our mechanistic understanding of EV-mediated delivery but also highlight scalable biomanufacturing strategies that position EVs as promising biofunctional excipients for more targeted (e.g., tumor-homing), and thus more effective, intracellular drug delivery (Contribution 10).

## 3. Conclusion and Future Perspectives

As shown in this SI, specialized additives known as biofunctional excipients are used in pharmaceutical formulations to improve the efficacy and performance of active pharmaceutical ingredients (APIs). Biofunctional excipients actively alter the properties of drugs, in contrast to standard excipients, which are usually inert and are generally employed to facilitate the manufacturing process or enhance the flavor and texture of medications.

The complex in vivo behavior of pharmaceutical additives which were once thought of as biologically inert excipients is becoming more widely acknowledged. As a result of this improved understanding, biofunctional excipients have emerged, concentrating on the biological behavior of the medicine as opposed to the processing and manufacturing of dosage forms. Smart polymers, lipids, and other safe, natural additives that can alter medication solubility, release kinetics, permeability, and pharmacokinetic characteristics are just a few examples of these recently developed biofunctional excipients. The biofunctional uses of a variety of excipients, such as polymers, smart polymers, bioadhesive excipients, lipids, cyclodextrins, surfactants, and pH-modifiers, were thoroughly covered in this review. Self-assembled polymeric, lipid, and surfactant nanosystems were made possible by these biofunctional excipients, and they may offer novel solutions to the inherent issues in biopharmaceutics, such as low permeability and solubility.

Future studies must address the biofates and regulatory issues of these biofunctional excipients, especially those that are biocompatible, biodegradable, and adaptable to certain medications or delivery methods. One interesting avenue is the investigation of smart polymers and sophisticated medication delivery systems that react to environmental cues. Furthermore, there may be room for innovation in the integration of biofunctional excipients with nanotechnology or customized medicine techniques.

To maximize the efficacy and safety of medications in clinical settings, future research should encourage better comprehension of the molecular pathways through which these excipients interact with medications. Moreover, in-depth studies should be conducted to identify and discover the potentials, and particularly undesirable biofunctions, such as changes in metabolism, elimination, and distribution. Excipient interactions with enzymes, proteins, carriers, cells, and other biological components are currently widespread and have the potential to negatively impact therapeutic functioning or cause unintended and inexplicable adverse effects.
